# Altered Intracerebellar Functional Connectivity in Friedreich’s Ataxia: A Graph-Theory Functional MRI Study

**DOI:** 10.1007/s12311-025-01785-3

**Published:** 2025-01-14

**Authors:** Mario Tranfa, Teresa Costabile, Giuseppe Pontillo, Alessandra Scaravilli, Chiara Pane, Arturo Brunetti, Francesco Saccà, Sirio Cocozza

**Affiliations:** 1 Department of Advanced Biomedical Sciences, University of Naples “Federico II”, Via Pansini 5, 80131 Naples, Italy; 2 Department of Clinical and Experimental Medicine, “Luigi Vanvitelli” University, Naples, Italy; 3 Department of Neurosciences and Reproductive and Odontostomatological Sciences, University of Naples “Federico II”, Naples, Italy

**Keywords:** Friedreich’s Ataxia, Functional MRI, Graph theory, Cognitive impairment, Verbal memory

## Abstract

Historically, Friedreich’s Ataxia (FRDA) has been linked to a relatively preserved cerebellar cortex. Recent advances in neuroimaging have revealed altered cerebello-cerebral functional connectivity (FC), but the extent of intra-cerebellar FC changes and their impact on cognition remains unclear. This study investigates intra-cerebellar FC alterations and their cognitive implications in FRDA. In this cross-sectional, single-center study, resting-state functional MRI data from 17 patients with FRDA (average age 27.7 ± 13.6 years; F/M = 6/11) and 20 healthy controls (HC) (average age 29.4 ± 9.7 years; F/M = 9/11), all of whom underwent neuropsychological testing, were analyzed. From functional connectivity matrices, graph measures were computed at both the network and node levels using two complementary parcellations. FRDA patients exhibited decreased global efficiency (*p* = 0.04), nodal degree (*p* = 0.001) and betweenness centrality (*p* = 0.04) in the vermal portion of lobule VIII, along with reduced global efficiency in cerebellar regions belonging to the Control-A network (*p* = 0.02), one of the three subdivisions of the Frontoparietal network. Verbal memory deficits correlated with global efficiency in both the vermal portion of lobule VIII (*r* = 0.53, *p* = 0.02) and the cerebellar regions of the Control-A network (*r* = 0.49, *p* = 0.05). Graph analysis revealed regional intra-cerebellar FC changes in FRDA, marked by reduced functional centrality in cerebellar regions of the vermis and responsible for executive functions. These changes correlated with cognitive alterations, highlighting the role of the cerebellar cortex in the cognitive impairment observed in FRDA.

## Introduction

Friedreich’s Ataxia (FRDA), caused by an expansion of an abnormal trinucleotide intronic GAA triplet in the FXN gene on chromosome 9 [1], represents the most common cause of hereditary ataxia [2], a large spectrum of conditions that are almost unequivocally related to the presence of a marked and significant involvement of the cerebellar cortex [3]. Nevertheless, at least from an imaging perspective, FRDA represents an interesting and possibly unique model of disease, given that the degree, and significance, of cerebellar cortical damage in its pathophysiology is still highly debated. Indeed, although a paradigm shift has been observed in FRDA, moving from the classical neuroimaging vision of a spared cerebellum [4] to the demonstration, via advanced MRI techniques, of a relatively significant involvement of this region [5], information about the extent and relevance of these changes is still needed.

This is particularly true with reference to the functional counterparts of the relatively wide knowledge about the structural alterations occurring in this condition [6–8]. Indeed, only few data about functional connectivity (FC) alterations in FRDA are available in literature. Previous papers reported evidence of altered FC assessed through the application of resting state functional MRI (RS-fMRI) methods [9, 10], with changes in the brain intrinsic functional architecture encompassing both intra-cerebral and cerebello-cerebral connections, that have been also recently described by means of magnetoencephalography (MEG) [11, 12]. These lines of evidence, highlighting a cerebellar involvement in a wide range of FC changes in FRDA, leave unanswered the question about possible cerebellar regional network alterations.

Moreover, while the hypothesis of a cerebellar involvement in the development of FRDA-related cognitive impairment is gaining increasing interest in the scientific community, with recent evidence of a subtle, yet significant, cerebellar cortex volume loss characterizing these patients and impacting on different cognitive domains [8], direct evidence of the relationship between a possible aberrant intra-cerebellar FC profile and cognitive performances is still missing [13].

Here, using two different parcellations based on anatomical [14] and functional [15] segregation respectively, we investigated the possible presence of altered intra-cerebellar FC in FRDA via graph analysis approach to RS-fMRI data, along with its potential repercussions on cognition, to further expand our knowledge about the role of the cerebellum in this condition.

## Materials & Methods

### Participants

A comprehensive overview of demographic and clinical data of the study population is available in Table 1. Twenty-four FRDA patients (31.3 ± 15.0 years; F/M = 9/15) were enrolled at the outpatient clinic for movement disorder of University “Federico II” of Naples, Italy. Inclusion criteria were the diagnosis of FRDA with homozygous expansion of the FXN gene through conventional genetic test with short and long triplet repeat primed polymerase chain reaction, while exclusion criteria were the presence of any psychiatric disorder or excessive motion during the MRI scan (see *MRI data processing*). A group of 24 Healthy Controls (HC) of comparable age and sex was also recruited (30.7 ± 15.5 years; F/M = 9/15).

**Table 1:** Demographic and clinical data of the subjects included in the study

	FRDA	HC	*p*-value
Age (years)	31.3 ± 15.0	30.7 ± 15.5	0.79
Sex (F/M)	9/15	9/15	1.00
Education (years)	12.1 ± 2.9	12.5 ± 3.2	0.72
Age At Onset	16.6 ± 10.2	n.a.	
Disease Duration	10.9 ± 6.6	n.a	
SARA (median and range)	16.0 (7.0–32.0)	n.a	
GAA1	937 ± 696	n.a	
*GGA2*	1014 ± 495	n.a.	

Unless otherwise indicated, data are expressed as mean ± standard deviation

FRDA, Friedreich’s Ataxia; HC, healthy controls; GAA1, GAA triplet repeats minor allele; GAA2, GAA triplet repeats major allele; SARA, Scale for the Assessment and Rating of Ataxias; n.a., not applicable

Patients were tested with a complete neuropsychological battery (Table 2), whose complete description is available in [9] and underwent MRI brain scan within 1 week from the clinical assessment. Briefly, the battery comprised tests for global cognitive function (Montreal Cognitive Assessment - MOCA), language (Naming Nouns Test and Pointing Names Test), intelligence (Raven Colored Progressive Matrices), executive functions (Symbol Digit Modalities Test - SDMT, Trail Making Test - TMT, Brief Stroop Test, Phonetic and Semantic Fluencies), memory (Digit span, 10/36 Spatial Recall Test, Rey Auditory Verbal Learning Test - RAVLT) and visuospatial functions (Segment length discrimination, Mental rotation). Neuropsychological test scores were corrected for speech and upper limbs impairment according to [16].

**Table 2:** Neuropsychological battery scores

	FRDA	HC	*p*-value
10/36 Spatial Recall Test	18.5 (6.6)	22.9 (3.8)	**0.02**
10/36 Spatial Recall Test - Delayed	6.5 (2.4)	8.2 (1.8)	**0.01**
Rey Auditory Verbal Learning Test - Immediate Recall	40.8 (12.6)	47.5 (9.4)	**0.05**
Rey Auditory Verbal Learning Test - Delayed Recall	9.3 (3.9)	10.5 (3.4)	0.25
Segment Length Discrimination	26.9 (2.2)	28.5 (1.7)	**0.003**
Montreal Cognitive Assessment	22.3 (3.6)	26.2 (2.3)	**< 0.001**
Pointing Names Test	23.9 (0.3)	24 (0.2)	0.30
Naming Nouns Test	13.9 (1.3)	14.2 (1.2)	0.57
Raven Colored Progressive Matrices	29.6 (6.3)	33 (5.4)	**0.05**
Digit Span Test	7 (4.4)	6.5 (1.1)	0.45
Trail Making Test -A	67 (68.9)	29.8 (8.8)	**< 0.001**
Trail Making Test -B	127.3 (57.7)	73.2 (24.2)	**< 0.001**
Brief Stroop Test	50.1 (20.3)	26.9 (7.4)	**< 0.001**
Semantic Fluency Test	18.5 (5.8)	23.7 (4.9)	**0.001**
Phonemic Fluency Test	27.5 (11.7)	41.3 (8.8)	**< 0.001**
Mental Rotation Test	93.3 (15.9)	105.8 (4.7)	**0.008**
Symbol Digit Modalities Test	35.8 (10)	57.3 (13.6)	**< 0.001**
Attentional Matrices Test	50.5 (8.7)	55.5 (3.2)	0.12

Unless otherwise indicated, data are expressed as mean ± standard deviation

FRDA, Friedreich’s Ataxia; HC, healthy controls; MOCA, Montreal Cognitive Assessment; RCPM, Raven Colored Progressive Matrices; SPART, 10/36 Spatial Recall Test; SPART-D, SPART delayed; RAVLT, Rey Auditory Verbal Learning Test Immediate Recall; RAVLT-D, RAVLT Delayed Recall; SLD, Segment Length Discrimination; SDMT, Symbol Digit Modalities Test; TMT, Trail Making Test; n.a., not applicable

## Standard Protocol Approvals, Registrations, and Patient Consents

The study received prior approval from the “Carlo Romano” Ethical Committee at the University of Naples “Federico II” (Italy) (approval no. 47/15) and was conducted in accordance with the Declaration of Helsinki. Written informed consent was obtained from all participants.

## MRI Data Acquisition

MRI data were acquired on a 3 Tesla MR scanner (Trio, Siemens Medical Systems, Erlangen, Germany), including a volumetric T1-weighted sequence (MPRAGE; TR = 1900 msec; TE = 3.4 msec; TI = 900 msec; flip angle = 9°; voxel size = 1 × 1 × 1mm [3]; number of axial slices = 160) for anatomical coregistration and an Echo Planar Imaging (EPI) T2*-weighted acquisition (TR = 2500 msec; TE = 40 msec; 64 × 64 acquisition matrix; number of axial slices = 30; voxel size = 3 × 3 × 4 mm [3]; gap = 1 mm; 200 time points leading to a total acquisition time of 8’27”) for the RS-fMRI assessment. The participants were instructed to lay supine with their eyes closed, without falling asleep. Straps and foam pads were used to reduce head motion, and after the imaging session patients were asked if they were able to not fall asleep.

## MRI Data Processing

RS-fMRI data were processed with the CONN toolbox (v.21, http://www.nitrc.org/projects/conn) based on SPM12.

A complete list of all the preprocessing steps of RS-fMRI data here done is available in [9]. Briefly, preprocessing involved removing the first 5 volumes, correcting for motion and slice timing, temporal despiking, band-pass filtering (0.008–0.09 Hz), and spatial smoothing with a 6-mm Gaussian kernel. The motion correction was obtained by realigning each study’s volumes to the first volume, optimizing translation and rotation parameters to minimize a least-squares cost function based on the voxel-by-voxel intensity differences from the reference image [17].

The mean displacement at each time point was calculated as the root-mean-square (RMS) of the translation parameters. MRI scans were excluded from the analysis if they exhibited a mean relative RMS ≥ 0.20 [18], or if displacement exceeded 2.0 mm or 2.0 degrees of rotation along any axis. Additionally, time points with a framewise differential signal intensity greater than 9 z-scores were removed and replaced with the mean framewise value.

Functional data were registered to the standard MNI space, resampled to an isotropic voxel (2 × 2 × 2 mm [3]) and visually checked by an experienced neuroradiologist.

## MRI Data Analysis

Prior to the RS-fMRI data analysis, we excluded two FRDA patients who refused to undergo MRI assessment due to anxiety, two patients and four HC participants due to low image quality as assessed through a visual quality check, and three FRDA patients due to excessive motion. This resulted in a final sample of 17 FRDA patients (27.7 ± 13.6 years; F/M = 6/11) and 20 HC participants (29.4 ± 9.7 years; F/M = 9/11). For the study of the FC profile of the cerebellum, to test the hypothesis of a complex pattern of cerebellar involvement in these patients of both anatomically and functionally segregated regions, for each subject BOLD signal time course was calculated using two different cortical parcellations, namely 26 anatomical regions from the AAL3 atlas [14] and 17 RS-networks based region obtained from the functional parcellation by Buckner and colleagues [15]. For each atlas-defined region of interest (ROI), the corresponding correlation maps of the BOLD signal across the cerebellum were generated, considering the time courses of cerebrospinal fluid and white matter signals, and the six translations and rotations parameters along the three main axes from the spatial normalization.

As is standard procedure, the resulting FC matrices were thresholded using a fixed network cost level to keep only the strongest 20% of connections. Specifically, the physical cost of connecting the components of a spatially embedded network is often referred to as its wiring cost or network cost level. As connection density is considered a reliable proxy for this property, we used it to threshold the connectivity matrices before computing graph properties. This approach helps reduce the influence of spurious connections on network topology and ensures that the number of connections remains consistent across all subjects [19].

The following graph measures were then evaluated: global efficiency (defined as the average inverse shortest path length between a given node and all other nodes in the network), local efficiency (which measures the average inverse shortest path length between a given node and its neighbors), betweenness centrality (the fraction of all shortest paths including a given node), average path length (the average length of the paths between each pair of nodes), clustering coefficient (the fraction of triangles around a node) and nodal degree (the number of edges connected to a given node).

### Statistical Analysis

Possible differences in age and sex between FRDA patients and HC were assessed with an independent samples t-test and chi-square test, respectively, while possible between groups differences in terms of graph measures were evaluated via General Linear Model analysis, correcting for age, sex and the average RMS (to account for any residual movement effects). Results were considered statistically significant if p-value ≤ 0.05 after false discovery rate (FDR) correction for multiple comparisons. Prior to independent samples t-test, the assumption of equal variances was confirmed using Bartlett’s test (*p* = 0.17).

Finally, as an exploratory analysis, the metrics that proved to be significantly different between groups were tested for possible correlation with the neuropsychological status via Pearson correlation coefficient. Given the exploratory nature of this additional analysis, p-values of Pearson correlation coefficients were considered significant for *p* ≤ 0.05 and not corrected for multiple comparisons [20].

Statistical analyses for the RS-fMRI data were performed within CONN, while correlations with clinical data were probed using RStudio (version 4.2.1).

### Data Availability Statement

All data generated during the current study can be made available upon reasonable request from the corresponding author.

## Results

No differences between FRDA patients and HC emerged in terms of age (*p* = 0.79) or sex (*p* = 1.00).

When evaluating possible differenced in terms of neuropsychological data, FRDA patients showed a relatively wide range of alterations in cognitive tests compared to HC (Table 2), including but not limited to the MOCA test (*p* < 0.001), the SDMT, *p* < 0.001), Semantic (*p* = 0.001) and Phonemic Fluency (*p* < 0.001) and the RAVLT - Immediate Recall (*p* = 0.05).

The two subgroups that underwent MRI assessment also did not differ in terms of age (*p* = 0.68) or sex (*p* = 0.55), while mean motion was significantly higher in FRDA compared to HC (0.05 ± 0.02 vs. 0.02 ± 0.01, *p* < 0.001).

**Fig. 1:**
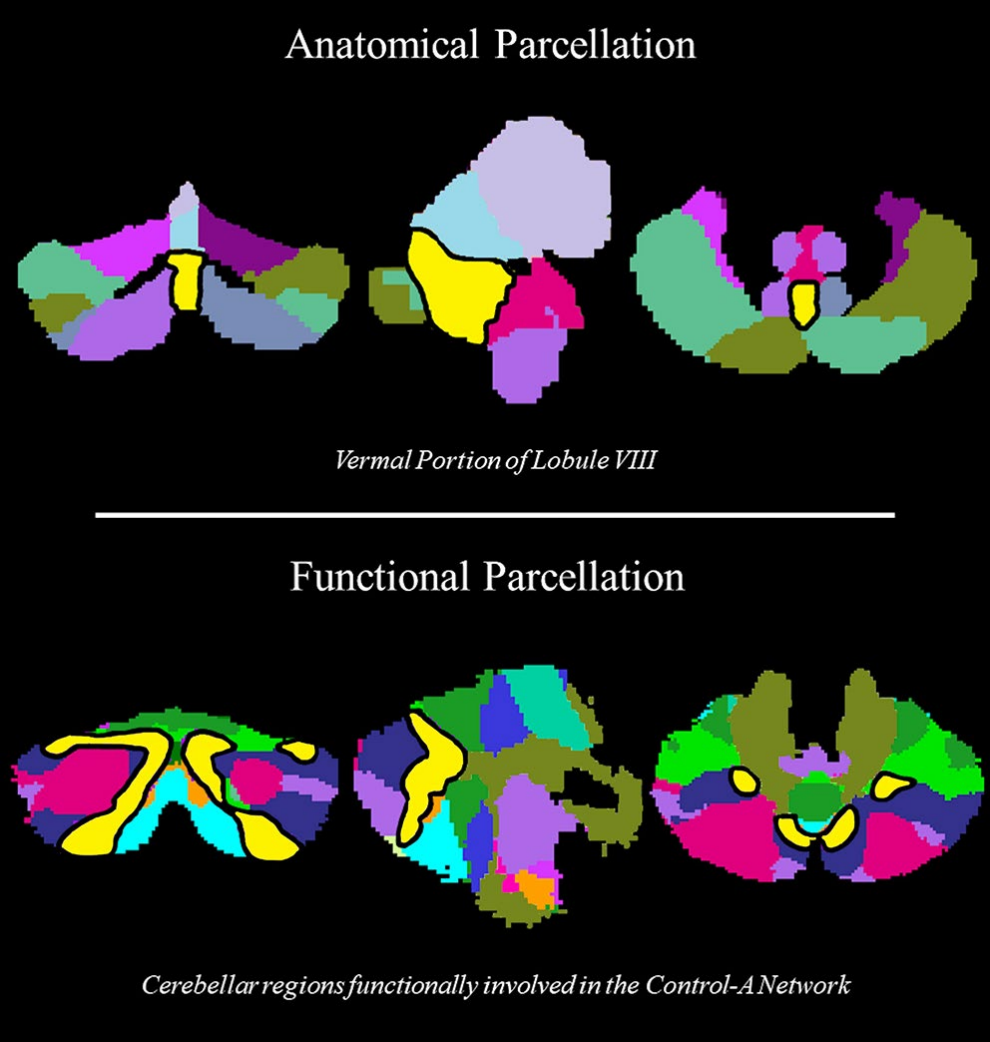
**Cerebellar areas that differed between FRDA and HC.** Cerebellar areas (yellow regions in black demarcation line) that proved to differ in terms of graph-derived measures between FRDA and HC. In the upper row, the segmentation according to the AAL3 atlas [14] showing the presence of significant differences at the level of the vermal portion of lobule VIII, while in the lower row the segmentation according to the Buckner atlas [15] showing significant differences at the level of areas that are functionally involved in the Control-A network. FRDA, Friedreich’s Ataxia; HC, healthy controls; AAL3, automatic anatomical labelling 3.

When performing the FC analysis using the anatomical parcellation, FRDA patients showed a significant alteration in different graph metrics at the level of the vermal portion of the lobule VIII compared to HC. In particular, FRDA patients showed a reduction compared to HC of the global efficiency of this region (*p* = 0.04), coupled to a reduction in betweenness centrality (*p* = 0.04) and nodal degree (*p* = 0.001) (Fig. 1). No increases in graph measures were found when probing the inverse contrast. Interestingly, the analysis performed using the functional parcellation showed a similar significant reduction of the global efficiency in FRDA patients compared to HC at the level of cerebellar areas functionally recruited in the Control-A network (*p* = 0.02) (Fig. 1), part of the Frontoparietal-Control Network and encompassing the paravermal areas of the posterior lobe of the cerebellum. Similarly to the analysis performed using the anatomical parcellation, no increase in graph measures were found in FRDA patients. A complete list of the between groups differences for the two analyses can be found in Table 3.

**Table 3:** Differences in RS-fMRI derived graph measures between FRDA patients and HC

Regions	Graph Measures	Effect size	*p*-value
Vermal portion of lobule VIII			
	Global efficiency	*0.17*	0.04
	Betweenness centrality	*0.03*	0.04
	Nodal degree	*3.90*	0.001
Control-A network			
	Global efficiency	*0.24*	0.02

FRDA, Friedreich’s Ataxia; HC, healthy controls; RS-fMRI, resting state functional MRI

**Fig. 2:**
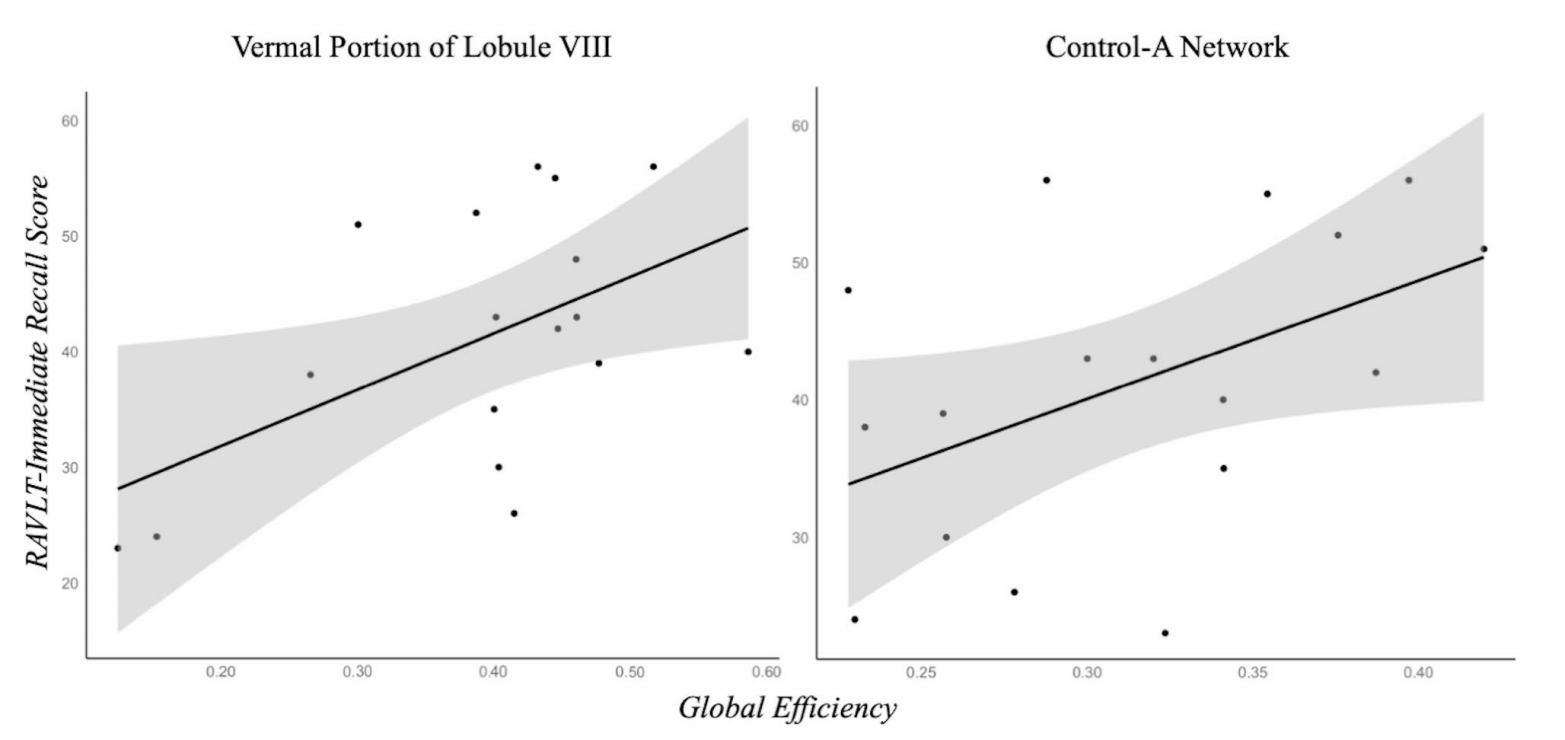
**Association between verbal memory and global efficiency.** Scatterplots showing the relationship between verbal memory functions, assessed via the RAVLT-immediate recall scores, and graph measures of global efficiency of the vermal portion of the Lobule VIII (left) and cerebellar areas of the Control-A network (right). RAVLT, Rey Auditory Verbal Learning Test.

Finally, the correlation analysis between altered intra-cerebellar functional metrics and cognitive scores in FRDA showed the presence of a significant correlation between verbal memory, assessed with the RAVLT immediate recall scores, and the altered measures of reduced global efficiency for both the anatomical vermal portion of the lobule VIII (*r* = 0.53, *p* = 0.02, CI = 0.07–0.81) and the functional area of the CON-A network (*r* = 0.49, *p* = 0.05, CI = 0.01–0.79) (Fig. 2).

## Discussion

In this study we explored the intra-cerebellar FC profile of FRDA patients with the application of a graph analysis approach on RS-fMRI derived connectivity matrices computed with both anatomical and functional parcellations, to investigate this different and possibly complementary information. Differently from previous RS-fMRI studies, that explored alterations in functional connectivity with a whole-brain seed-to-voxel approach [9, 10], our study focused on short range (i.e., intra-cerebellar) connections, a feature not yet explored in this condition. Following previous studies highlighting subtle, yet detectable, alterations in cerebellar micro- [6] and macrostructure [8], we explored possible alterations in intra-cerebellar connectivity by summarizing relevant intra-cerebellar functional properties, encompassing measures of network integration and segregation. Our results showed, for the first time, that reduced intra-cerebellar functional integration is detectable in FRDA patients compared to HC, and that these alterations can at least in part explain the cognitive changes observed in the disease.

While historically the cerebellum was thought to be only linked to motor functions, it is now well known that this region is in functional connection with cerebral areas responsible for a wide range of cognitive functions, including but not limited emotional and language processing, working memory and executive functions [21, 22]. Indeed, many lines of evidence suggest that through its connections with prefrontal and associative temporo-parietal areas, the cerebellum could encode internal models to monitor and balance mental activities [23].

Given this background, it is easy to hypothesize and worth exploring its possible functional involvement in determining, at least in part, the observed cognitive alterations in FRDA patients. Some information about altered cerebello-cerebral functional connections in these patients, probed via RS-fMRI, are available in literature [9, 10]. In particular, a seed-based study showed that FRDA is characterized by decreased FC between the middle frontal gyrus and the cerebellum at the level of the lobule VI and the vermis, along with increased intra-cerebral functional connections possibly representing compensatory mechanisms [9]. These results have been recently expanded in another seed-based study reporting decreased FC between the pre- and postcentral cortex and the anterior cerebellum, as well as between the prefrontal cortex and the posterior cerebellum in FRDA [10]. Nevertheless, no information about intra-cerebellar FC changes measured via RS-fMRI analysis has been reported in FRDA to date. In investigating these aspects, extracting and analyzing graph measures to summarize network properties reduces the number of tests and increases the statistical power in identifying subtle connectivity alterations, especially in the context of rare diseases, where small samples are inevitable.

Our main result is that using either an anatomical or a functional parcellation of the cerebellum, therefore investigating the possible different contribution of distinct cerebellar areas, we were able to find a pattern of reduced intra-cerebellar connectivity in FRDA patients. In particular, the vermal portion of the Lobule VIII showed a significant reduction in global efficiency, betweenness centrality and nodal degree, overall highlighting a reduction in centrality of this region.

Similarly, a pattern of reduced global efficiency was observed in cerebellar regions functionally related to the Control-A network, a subdivision of the Frontoparietal Network, responsible for executive functions and encompassing the postero-medial portion of the cerebellar hemispheres [15].

When testing possible correlations between altered intra-cerebellar functional connections and the cognitive status of FRDA patients, we observed a correlation between altered graph measures and altered verbal memory and language processing performances. This observation, aside indirectly confirming that advanced MRI techniques such as RS-fMRI might be indeed capable of capturing FRDA-related cerebellar alterations, also suggest that these latter could impact on patients’ cognitive performance. Indeed, although there is evidence supporting the link between vermal portion of lobule VIII and timing and coordination of horizontal smooth pursuit eye movements, and by extension visual perception [24], it has also been reported that the posterior part of the vermis (including the lobule VIII portion) plays a role in attentional impairment in patients with attention deficit hyperactivity disorder (ADHD) [25]. As attention and verbal working memory are strictly related cognitive domains [26], with evidence of cerebellar integrity being indispensable for proper verbal working memory functioning [27], it is possible to speculate that its functional alteration could have an impact on verbal information storage and manipulation during goal-directed tasks. This hypothesis is supported by previous findings showing decreased FC between the vermis and middle frontal gyrus [9], which is a hub region containing mixed neuronal populations connected with regions belonging to both Dorsal and Ventral Attention networks [28], involved in goal-directed and stimulus-driven attention processes, respectively [29].

Nonetheless, we did not find a significant association between the Attentional Matrices Test scores and altered graph measures. A possible explanation could be that the relationship between task-directed attention and an altered FC profile of the vermal portion of the lobule VIII might be more specific for verbal memory tasks. On the other hand, our results, showing that verbal memory performance and FC changes of the Control-A network are significantly related, also suggest that the relationship between a localized alteration and cognition could be too simplistic. Indeed, the activation of the Control-A network is known to be indispensable for accurate and flexible adaptive control during goal-directed actions [29] and subsequently for verbal working memory related tasks [30].

Taken all together, these observations suggest that functional integrity of both the vermal portion of the lobule VIII and the Control-A network, along with their possible interplay, could be a key component of verbal memory processing alterations in FRDA patients, although longitudinal studies are needed to shed light on the temporal, and maybe causative, relationship between these alterations. Furthermore, as a previous study from our group identified a cluster of reduced volume only in lobule IX using voxel-based morphometry [8], it is possible to speculate that the functional changes here observed may be spatially independent of their structural counterparts. Nonetheless, further studies with larger sample sizes, and preferably longitudinal designs, are required to disentangle the temporal and spatial association between structural and functional cerebellar alterations in FRDA.

Obviously, this study does not come without limitations. First, the relatively small sample size, which is a typical trade-off of rare diseases such as FRDA, might have limited our possibility to observe even larger and stronger changes in intra-cerebellar connectivity changes occurring in these patients. Second, since their exploratory nature, the correlation tests between FC and cognitive performance were not corrected for multiple comparisons. For this reason, our consideration about the clinical counterpart of the observed FC changes should be interpreted as speculative. Furthermore, while other imaging biomarkers, such as cerebellar white matter disruption [6] or dentate nucleus integrity [31], have been shown to correlate more strongly with clinical severity, altered functional changes are more subtle in FRDA and appears to have weaker clinical relevance, possibly reflecting a lesser degree of alteration compared to other brain properties. It is also worth noting that, while providing complementary information, our results demonstrated the occurrence of a spatial heterogeneity of effects across different parcellations, further highlighting an intrinsic limitation of atlas-based approaches (namely, that such methods can yield substantially different results depending on the choice of the parcellation). Therefore, further studies leveraging data from larger cohorts, such as those recently available in multi-center studies are needed to better characterize these subtle alterations. Such efforts could also involve applying more fine-grained cerebellar parcellations [32], further overcoming the limitations of possibly obscuring meaningful biological effects in cases of small samples or effect sizes.

## Conclusion

In conclusion, our observations confirm that the cerebellum is involved in the pathophysiology of FRDA not only from a structural, but also from a functional standpoint, and suggests that integrating information from different parcellations could provide complementary knowledge and help us in decoding the exact relationship between FC alterations and cognitive changes in FRDA.

## Data Availability

The data that support the findings of this study are available on request from the corresponding author.
